# Draft Genome Sequence of an Invasive Multidrug-Resistant Strain, *Pseudomonas aeruginosa* BK1, Isolated from a Keratitis Patient

**DOI:** 10.1128/genomeA.00153-14

**Published:** 2014-03-27

**Authors:** Lakshmi Priya Jeganathan, Logambiga Prakash, Neelamegam Sivakumar, Aju Antony, Sami Alqarawi, Lalitha Prajna, Bharanidharan Devarajan, Vidyarani Mohankumar

**Affiliations:** a Department of Ocular Microbiology, Aravind Medical Research Foundation, Madurai, India; b Department of Bio-informatics, Aravind Medical Research Foundation, Madurai, India; c BioSciences Core Lab, King Abdullah University of Science and Technology, Thuwal, Saudi Arabia

## Abstract

*Pseudomonas aeruginosa* infections are difficult to treat due to the presence of a multitude of virulence factors and antibiotic resistance. Here, we report the draft genome sequence of *P. aeruginosa* BK1, an invasive and multidrug-resistant strain, isolated from a bacterial keratitis patient in southern India.

## GENOME ANNOUNCEMENT

*Pseudomonas aeruginosa* is a Gram-negative bacterium capable of causing a variety of life-threatening human infections. The metabolically versatile *P. aeruginosa* is an opportunistic pathogen of plants, animals, and humans and is ubiquitously distributed in soil and aquatic habitats (1). Since most of its strains are highly virulent, *P. aeruginosa* ulcers are generally more difficult to treat and result in worse visual outcomes than other bacterial ulcers.

In developing countries, ocular trauma remains a major risk factor for encountering ocular *P. aeruginosa* infections, whereas in developed countries, it is frequently associated with contact lens wear (2, 3). An array of virulence factors contribute to the pathogenicity of *P. aeruginosa*. Cell-associated structures, including flagella, pili, fimbriae, and endotoxin (lipopolysaccharide), as well as extracellular products, such as proteases and exotoxins, are associated with virulence, invasiveness, and colonization (4). Also, clinical isolates of *Pseudomonas* often exhibit multiple resistances to antibiotics. Multidrug resistance is often related to the presence of specific efflux pumps and porins in *P. aeruginosa* strains (5). Hence, to efficiently control *P. aeruginosa* infections, it is mandatory to understand the intrinsic and extrinsic virulence mechanisms of this bacterium.

We announce here the draft genome sequence of the *P. aeruginosa* strain BK1, isolated from a keratitis patient at Aravind Eye Hospital, Madurai, India. The strain was resistant to broad-spectrum antibiotics, like moxifloxacin, levofloxacin, cefotaxime, and ciprofloxacin. The invasive nature of the strain was confirmed by studying the expression of the type III secretion effector molecules encoded by *exoU*, *exoS*, and *exoT* by PCR and Western blot analysis. The strain had an *exoST* genotype and an ExoT phenotype and was highly capable of invading the corneal epithelial cells *in vitro*.

Genomic DNA from *P. aeruginosa* BK1 was isolated using the QIAamp DNA minikit from Qiagen (Hilden, Germany). The strain identity was then confirmed to the species level by sequencing the 16S rRNA region. Whole-genome sequencing was performed using the Ion Torrent (PGM) sequencer with 400-bp read chemistry (Life Technologies). Sequencing was carried out as per the Ion 318 Chip sequencing protocol. The data were filtered with a Phred score of >20, and the filtered sequences were assembled *de novo* using the CLC Genomics Workbench software version 6.5.1 (CLC bio, Germantown, MD). The minimum contig size was set to 500 nucleotides, which generated 163 contigs, with an estimated genome size of 6,451,158 bp. In total, 6,736 protein-encoding genes and 61 RNA-encoding genes were annotated using the RAST server. Comparative genome analysis was performed with the *P. aeruginosa* PAO1 genome (GenBank accession no. NC_002516.2) using MAUVE (6). While 97% of the PAO1 genome aligned with the BK1 genome, 425,466 bp in the BK1 genome showed no homology, which includes the *P. aeruginosa* genomic islands (PAGI) 1, 4, 5, and 10.

### Nucleotide sequence accession numbers.

This whole-genome shotgun project has been deposited at DDBJ/EMBL/GenBank under the accession no. JBTQ00000000. The version described in this paper is the first version, JBTQ01000000.
